# Supplementary material for: Description of the new species Sigambra nkossa (Annelida, Pilargidae), with an analysis of the distribution patterns of polychaetes associated with artificially hydrocarbon-enriched bottoms
Source: PeerJ. 2022 Oct 19;10:e13942. doi: 10.7717/peerj.13942 (PMC9587720; doi:10.7717/peerj.13942)
Supplement: Table S1 — Snk: Sigambra nkossa sp. nov.; Sp: Sigambra parva; Csp: Capitella sp. Pt: Paramphinome trionyx. Rsp: Raricirrus sp. Ob: Oxydromus berrisfordi. Ls: Lindaspio sebastiena. Amp: Ampharetidae sp.; DDP: distance from drilling point (m); HYD: Total hydrocarbons (mg/kg of sediment); Ba: Barium (mg/kg of sediment); CS: coarse sand (%); S&C: silt and clay (%); PW: pore water (%); OM: organic matter (%); N: Nitrogen (%); P: Phosphorous (%). [file peerj-10-13942-s001.docx]

| Station | YEAR | Snk | Sp | Csp | Pt | Rsp | Ob | Ls | Amp | DDP | HYD | Ba | CS | S&C | PW | OM | N | P |
| --- | --- | --- | --- | --- | --- | --- | --- | --- | --- | --- | --- | --- | --- | --- | --- | --- | --- | --- |
| 6 | 2000 | 247 | 0 | 410 | 0 | 68 | 12 | 9 | 0 | 70 | 166000 | 146 | 23 | 37.6 | 40.7 | 11.9 | 0.2 | 1010 |
| 5 | 2000 | 485 | 0 | 278 | 3 | 69 | 8 | 0 | 5 | 175 | 29900 | 85.2 | 24.3 | 44 | 44.8 | 11 | 0.181 | 1000 |
| 4 | 2000 | 415 | 1 | 30 | 16 | 24 | 0 | 0 | 2 | 250 | 9520 | 31.3 | 44.7 | 38.5 | 49.9 | 11.3 | 0.166 | 1300 |
| 7 | 2000 | 22 | 12 | 25 | 35 | 2 | 2 | 0 | 0 | 150 | 623 | 5.05 | 29.1 | 67.4 | 40.6 | 10.1 | 0.133 | 1670 |
| 1 | 2000 | 0 | 0 | 0 | 1 | 0 | 0 | 0 | 0 | 4000 | 18 | 0.281 | 39.6 | 47.5 | 53.3 | 13 | 0.05 | 1570 |
| 3 | 2000 | 7 | 9 | 6 | 9 | 1 | 0 | 0 | 0 | 500 | 14 | 2.12 | 44.6 | 40.9 | 44.8 | 11.8 | 0.163 | 1920 |
| 2 | 2000 | 0 | 0 | 0 | 3 | 0 | 0 | 0 | 0 | 2000 | 10 | 0.172 | 60.6 | 34.7 | 41 | 10.3 | 0.247 | 1710 |
| 8 | 2000 | 0 | 1 | 2 | 4 | 1 | 0 | 0 | 0 | 300 | 10 | 0.344 | 23.6 | 68.5 | 43.4 | 11.6 | 0.14 | 1730 |
| 9 | 2000 | 0 | 1 | 0 | 1 | 0 | 0 | 0 | 0 | 600 | 10 | 0.247 | 39.6 | 53.3 | 42.7 | 10.6 | 0.107 | 1570 |
| 10 | 2000 | 0 | 1 | 0 | 5 | 0 | 0 | 0 | 0 | 2000 | 10 | 0.473 | 35.6 | 52.7 | 43.3 | 10.8 | 0.132 | 1750 |
| 11 | 2000 | 0 | 0 | 0 | 3 | 0 | 0 | 0 | 0 | 5300 | 10 | 0.161 | 48.5 | 44.1 | 42.1 | 11.2 | 0.157 | 1700 |
| 6 | 2002 | 635 | 0 | 2 | 14 | 0 | 5 | 0 | 0 | 70 | 64305 | 142 | 39.55 | 45.4 | 46.5 | 11.2 | 0.18 | 996 |
| 13 | 2002 | 1145 | 0 | 222 | 88 | 7 | 27 | 0 | 0 | 100 | 11400 | 71.8 | 0.45 | 78.7 | 48.4 | 10.4 | 0.18 | 1300 |
| 4 | 2002 | 271 | 4 | 305 | 300 | 2 | 3 | 2 | 27 | 230 | 1345 | 42.3 | 45 | 34.1 | 52.6 | 10.5 | 0.19 | 1342 |
| 3 | 2002 | 0 | 7 | 3 | 2 | 0 | 0 | 0 | 0 | 470 | 90 | 3.86 | 58.18 | 25.6 | 49.7 | 9.9 | 0.16 | 1900 |
| 15 | 2002 | 1 | 7 | 21 | 9 | 0 | 0 | 0 | 0 | 180 | 55 | 7.62 | 40.91 | 38.9 | 54.1 | 10.3 | 0.19 | 1898 |
| 16 | 2002 | 0 | 6 | 0 | 5 | 0 | 0 | 0 | 0 | 730 | 21 | 1.43 | 50 | 35.7 | 48.3 | 9 | 0.15 | 1890 |
| 2 | 2002 | 0 | 4 | 0 | 8 | 0 | 0 | 0 | 0 | 2000 | 10 | 0.604 | 38.18 | 43.3 | 54.8 | 11.3 | 0.19 | 1574 |
| 6 | 2003 | 1356 | 0 | 24 | 44 | 0 | 1 | 36 | 1 | 70 | 109000 | 94.6 | 24.3 | 27.3 | 35.3 | 18.4 | 0.14 | 778 |
| 13 | 2003 | 863 | 4 | 1028 | 146 | 0 | 0 | 1 | 207 | 100 | 9970 | 77.6 | 1.2 | 70.3 | 42.5 | 11.3 | 0.19 | 522 |
| 4 | 2003 | 650 | 6 | 743 | 0 | 0 | 0 | 0 | 53 | 230 | 8815 | 56.3 | 0 | 89.9 | 45.8 | 11.3 | 0.19 | 1020 |
| 18 | 2003 | 294 | 15 | 956 | 158 | 0 | 0 | 0 | 260 | 70 | 2667 | 36 | 0 | 79.4 | 48.5 | 12 | 0.25 | 824 |
| 19 | 2003 | 0 | 2 | 0 | 0 | 0 | 0 | 0 | 0 | 250 | 84.6 | 9.76 | 0 | 80.1 | 48.4 | 11 | 0.19 | 1461 |
| 3 | 2003 | 0 | 1 | 1 | 6 | 0 | 0 | 0 | 0 | 470 | 76.6 | 5.34 | 0.1 | 71.6 | 49.8 | 12.5 | 0.16 | 863 |
| 20 | 2003 | 0 | 0 | 0 | 0 | 0 | 0 | 0 | 0 | 500 | 40.2 | 1.27 | 12.7 | 50.6 | 43.6 | 12 | 0.15 | 1741 |
| 16 | 2003 | 0 | 1 | 0 | 4 | 0 | 0 | 0 | 0 | 730 | 29.8 | 1.53 | 17.6 | 48.4 | 47.9 | 11.3 | 0.18 | 810 |
| 17 | 2003 | 0 | 1 | 0 | 2 | 0 | 0 | 0 | 0 | 380 | 18.1 | 3.48 | 40.4 | 43.1 | 44.8 | 12.8 | 0.16 | 770 |
| 2 | 2003 | 0 | 0 | 0 | 1 | 0 | 0 | 0 | 2 | 2000 | 10 | 0.482 | 1.8 | 68.8 | 48.6 | 12.9 | 0.19 | 1023 |
