# Supplementary material for: Description of the new species Sigambra nkossa (Annelida, Pilargidae), with an analysis of the distribution patterns of polychaetes associated with artificially hydrocarbon-enriched bottoms
Source: PeerJ. 2022 Oct 19;10:e13942. doi: 10.7717/peerj.13942 (PMC9587720; doi:10.7717/peerj.13942)
Supplement: Table S2 — DC: dorsal cirri; VC: ventral ciri. [file peerj-10-13942-s002.docx]

| Specimen | Number of chaetigers | Total length (µm) | Width chaetiger 15 with parapodia (µm) | Width chaetiger 15 without parapodia (µm) | Dry weight (mg) | Length of prostomium (µm) | Width of prostomium (µm) | Length median antennae (µm) | Length lateral antennae (µm) | Dorsal peristomial cirri (µm) |
| --- | --- | --- | --- | --- | --- | --- | --- | --- | --- | --- |
| 1 | 134 | 26000 | 1900 | 850 | 2.1 | 420 | 750 | 1100 | 580 | 1280 |
| 2 | 23 | 2850 | 910 | 400 | 0.91 | 225 | 420 | 490 | 250 | 480 |
| 3 | 42 | 4450 | 810 | 280 | 0.81 | 275 | 330 | 400 | 290 | 300 |
| 4 | 78 | 13200 | 1130 | 485 | 1.13 | 380 | 470 | 680 | 365 | 620 |
| 5 | 109 | 19370 | 1750 | 760 | 1.75 | 420 | 650 | 1000 | 580 | 970 |
| 6 | 52 | 6050 | 1020 | 380 | 1.02 | 280 | 440 | 550 | 330 | 510 |
| 7 | 24 | 2400 | 570 | 190 | 0.57 | 175 | 240 | 260 | 175 | 185 |
| 8 | 45 | 4650 | 785 | 290 | 0.785 | 230 | 360 | 410 | 230 | 280 |
| 9 | 110 | 18950 | 1850 | 600 | 1.83 | 540 | 770 | 1060 | 600 | 920 |
| 10 | 122 | 21670 | 1850 | 750 | 1.9 | 550 | 730 | 1080 | 560 | 830 |
| 11 | 123 | 23350 | 1750 | 650 | 1.73 | 420 | 700 | 950 | 620 | 980 |
| 12 | 103 | 18200 | 1530 | 550 | 1.53 | 320 | 670 | 1000 | 610 | 870 |
| 13 | 94 | 13030 | 1260 | 570 | 1.26 | 370 | 470 | 850 | 540 | 730 |
| 14 | 81 | 10980 | 1250 | 575 | 1.25 | 310 | 470 | 890 | 410 | 630 |
| 15 | 118 | 20100 | 1850 | 800 | 1.87 | 510 | 630 | 970 | 825 | 1190 |
| 16 | 72 | 9630 | 1480 | 660 | 1.48 | 435 | 560 | 760 | 570 | 920 |
| 17 | 125 | 20450 | 1650 | 800 | 1.65 | 470 | 640 | 1280 | 650 | 940 |
| 18 | 107 | 15300 | 1320 | 580 | 1.32 | 340 | 620 | 915 | 450 | 720 |
| 19 | 53 | 7140 | 1080 | 510 | 1.08 | 330 | 500 | 760 | 420 | 585 |
| 20 | 104 | 15800 | 1530 | 650 | 1.55 | 380 | 540 | 1100 | 470 | 820 |
| 21 | 108 | 19200 | 1800 | 760 | 1.8 | 300 | 670 | 1020 | 700 | 900 |

| Specimen | Ventral peristomial cirri (µm) | Length DC1 (µm) | Length DC2 (µm) | Length DC3 (µm) | Length VC1 (µm) | Length VC2 (µm) | Length anal cirri (µm) | Starting chaetiger hooks | Starting chaetiger spines | Starting chaetiger protruding aciculae tips |
| --- | --- | --- | --- | --- | --- | --- | --- | --- | --- | --- |
| 1 | 730 | 1850 | 450 | 600 | 380 | - | 2460 | 5 | 11 | 0 |
| 2 | 320 | 675 | 180 | 220 | 100 | - | 595 | 5 | 9 | 9 |
| 3 | 225 | 590 | 110 | 175 | 150 | - | 680 | 5 | 9 | 2 |
| 4 | 380 | 1080 | 230 | 325 | 175 | - | 1100 | 6 | 9 | 26 |
| 5 | 770 | 1450 | 320 | 350 | 320 | - | 1550 | 6 | 11 | 54 |
| 6 | 340 | 830 | 160 | 225 | 120 | - | 1130 | 5 | 9 | 7 |
| 7 | 170 | 320 | 85 | 120 | 70 | - | 440 | 5 | 9 | 1 |
| 8 | 200 | 470 | 90 | 135 | 75 | - | 710 | 5 | 9 | 4 |
| 9 | 530 | 1550 | 380 | 400 | 260 | - | 1150 | 5 | 9 | 23 |
| 10 | 620 | 1600 | 390 | 530 | 320 | - | 1390 | 5 | 9 | 48 |
| 11 | 680 | 1600 | 390 | 650 | 310 | - | 1800 | 5 | 9 | 0 |
| 12 | 760 | 1410 | 280 | 450 | 315 | - | 1225 | 5 | 9 | 52 |
| 13 | 570 | 1180 | 235 | 390 | 220 | - | 1270 | 5 | 9 | 17 |
| 14 | 475 | 1150 | 215 | 355 | 190 | - | 1370 | 5 | 9 | 15 |
| 15 | 760 | 1985 | 360 | 540 | 350 | - | 2200 | 5 | 9 | 74 |
| 16 | 610 | 1310 | 330 | 550 | 210 | - | 900 | 5 | 9 | 40 |
| 17 | 700 | 1450 | 340 | 400 | 320 | - | 1280 | 5 | 11 | 78 |
| 18 | 585 | 1180 | 250 | 340 | 170 | - | 940 | 5 | 9 | 20 |
| 19 | 380 | 1060 | 170 | 210 | 160 | - | 1040 | 5 | 9 | 37 |
| 20 | 680 | 1540 | 260 | 340 | 245 | - | 1450 | 5 | 11 | 23 |
| 21 | 780 | 1700 | 430 | 610 | 350 | - | 1370 | 6 | 9 | 58 |
