# Supplementary material for: Description of the new species Sigambra nkossa (Annelida, Pilargidae), with an analysis of the distribution patterns of polychaetes associated with artificially hydrocarbon-enriched bottoms
Source: PeerJ. 2022 Oct 19;10:e13942. doi: 10.7717/peerj.13942 (PMC9587720; doi:10.7717/peerj.13942)
Supplement: Table S3 — (A) Results of the Parametric Multidimensional analysis for the environment descriptors. (B) Results of the Parametric Multidimensional analysis for the species abundance according to the sampling years. (C) Results of the Pearson correlation analysis between species abundance and environment descriptors. Significant differences/correlations are highlighted in bold. MD: Mahalanobis distance; Pearson: correlation coefficient; p: significance level; DDP: Distance from drilling points; HYD: Total hydrocarbons; Ba: Barium; CS: Coarse sand; S&C: Silt and clay; PW: Pore water; OM: Organic matter; N: Nitrogen; P: Phosphorous. [file peerj-10-13942-s003.docx]

| **A** | 2000 | |  | 2002 | |  | **B** | 2000 | |  | 2002 | |
| --- | --- | --- | --- | --- | --- | --- | --- | --- | --- | --- | --- | --- |
|  | MD | p |  | MD | p |  |  | MD | p |  | MD | p |
| 2002 | 0.669 | 0.272 |  | - | - |  | 2002 | 3.165 | 0.343 |  | - | - |
| 2003 | 0.709 | 0.190 |  | 0.004 | 0.992 |  | 2003 | 2.944 | 0.267 |  | 5.763 | 0.086 |

| **C** | Correlation | DDP | HYD | Ba | CS | S&C | PW | OM | N | P |  |
| --- | --- | --- | --- | --- | --- | --- | --- | --- | --- | --- | --- |
| *S. nkossa* sp. nov. | Pearson | -0.363 | **0.469** | **0.730** | -0.350 | 0.125 | -0.303 | **0.432** | 0.142 | **-0.541** |  |
|  | p | 0.058 | **0.05** | **<0.0001** | 0.068 | 0.525 | 0.117 | **0.022** | 0.470 | **0.003** |  |
| *S. parva* | Pearson | -0.285 | -0.261 | -0.182 | -0.019 | 0.194 | 0.171 | -0.268 | 0.245 | 0.165 |  |
|  | p | 0.142 | 0.179 | 0.355 | 0.925 | 0.322 | 0.386 | 0.168 | 0.209 | 0.402 |  |
| *Capitella* sp. | Pearson | -0.295 | 0.139 | **0.439** | **-0.505** | **0.456** | -0.071 | -0.020 | **0.457** | **-0.537** |  |
|  | p | 0.128 | 0.481 | **0.020** | **0.006** | **0.015** | 0.781 | 0.920 | **0.015** | **0.003** |  |
| *P. trionyx* | Pearson | -0.242 | -0.052 | 0.223 | -0.137 | 0.082 | 0.163 | -0.039 | 0.301 | -0.274 |  |
|  | p | 0.214 | 0.792 | 0.255 | 0.487 | 0.677 | 0.406 | 0.845 | 0.120 | 0.159 |  |
| *Raricirrus* sp. | Pearson | -0.200 | **0.596** | **0.558** | -0.035 | -0.195 | -0.170 | -0.028 | 0.156 | -0.220 |  |
|  | p | 0.307 | **0.001** | **0.002** | 0.860 | 0.320 | 0.387 | 0.889 | 0.428 | 0.260 |  |
| *O. berrisfordi* | Pearson | -0.226 | 0.368 | **0.540** | -0.244 | 0.147 | -0.035 | -0.114 | 0.138 | -0.148 |  |
|  | p | 0.247 | 0.054 | **0.003** | 0.211 | 0.456 | 0.859 | 0.562 | 0.483 | 0.452 |  |
| *L. sebastiena* | Pearson | -0.159 | **0.665** | **0.422** | -0.047 | -0.315 | **-0.506** | **0.811** | -0.093 | -0.303 |  |
|  | p | 0.420 | **0.000** | **0.025** | 0.814 | 0.102 | **0.006** | **< 0.0001** | 0.639 | 0.117 |  |
| Ampharetidae sp. | Pearson | **-0.387** | **0.431** | **0.497** | **-0.498** | 0.344 | 0.041 | 0.058 | **0.426** | **-0.554** |  |
|  | p | **0.042** | **0.022** | **0.007** | **0.007** | 0.073 | 0.835 | 0.769 | **0.024** | **0.002** |  |
