# Supplementary material for: Description of the new species Sigambra nkossa (Annelida, Pilargidae), with an analysis of the distribution patterns of polychaetes associated with artificially hydrocarbon-enriched bottoms
Source: PeerJ. 2022 Oct 19;10:e13942. doi: 10.7717/peerj.13942 (PMC9587720; doi:10.7717/peerj.13942)
Supplement: Table S4 — (A) ANOSIM pairwise tests (environmental descriptors). (B) Results of the Parametric Multidimensional Analysis for the sediment descriptors. (C) Results of the Parametric Multidimensional Analysis for the density of target species. (D) Results of the independent one-way ANOVAs for each sediment descriptor and target species. (E) Pairwise post-hoc comparisons (Tuckey test). Bold: Significant differences. NS: Non significant; R: R statistic; PP: Possible permutations; AP: Actual permutations; N: Number ≥ Observed; DDP: Distance from drilling point; HYD: Total hydrocarbons; Ba: Barium;CS: Coarse sand; S&C: Silt and clay; PW: Pore water; N: Nitrogen; P: Phosphorous; DF: degrees of freedom; SS: Sum of Squares; MS: Mean squares; F: Fisher’s test; p: significance level. [file peerj-10-13942-s004.docx]

| **A** | R | p | PP | AP | N |
| --- | --- | --- | --- | --- | --- |
| I vs. II | **0.444** | **0.012** | **330** | **330** | **4** |
| I vs. III | **0.989** | **0.001** | **5985** | **999** | **0** |
| II vs. III | **0.863** | **0.001** | **346104** | **999** | **0** |

| **B** | III | |  | II | |  | **C** | III | |  | II | |
| --- | --- | --- | --- | --- | --- | --- | --- | --- | --- | --- | --- | --- |
|  | MD | p |  | MD | p |  |  | MD | p |  | MD | p |
| II | **9.621** | **0.011** |  | - | - |  | II | **25.818** | **<0.0001** |  | - | - |
| I | **67.821** | **<0.0001** |  | **37.222** | **0.0003** |  | I | **35.059** | **<0.0001** |  | **19.637** | **0.0039** |

| **D** | DF | SS | MS | F | p |
| --- | --- | --- | --- | --- | --- |
| DDP | **2** | **4.655** | **2.328** | **17.329** | **<0.0001** |
| HYD | **2** | **53.722** | **26.861** | **177.048** | **<0.0001** |
| Ba | **2** | **12.821** | **6.411** | **63.591** | **<0.0001** |
| CS | 2 | 1.880 | 0.940 | 2.513 | 0.101 |
| S&C | **2** | **0.120** | **0.060** | **3.423** | **0.049** |
| PW | 2 | 0.009 | 0.004 | 2.675 | 0.089 |
| OM | 2 | 0.011 | 0.006 | 2.368 | 0.114 |
| N | 2 | 0.000 | 0.000 | 1.180 | 0.324 |
| P | **2** | **0.167** | **0.083** | **4.339** | **0.024** |
| *S. nkossa* sp. nov. | **2** | **43.195** | **21.597** | **179.281** | **<0.0001** |
| *Capitella* sp. | **2** | **25.918** | **12.959** | **39.718** | **<0.0001** |
| *P. trionyx* | **2** | **5.760** | **2.880** | **9.744** | **0.0007** |
| *O. berrisfordi* | **2** | **2.239** | **1.119** | **13.053** | **0.0001** |
| *Raricirrus* sp. | **2** | **2.897** | **1.448** | **6.879** | **0.004** |
| *L. sebastiena* | **2** | **1.335** | **0.668** | **8.175** | **0.002** |
| Ampharetidae sp. | **2** | **6.830** | **3.415** | **12.030** | **0.0002** |
| *S. parva* | **2** | **1.246** | **0.623** | **5.004** | **0.016** |
