# Supplementary material for: Description of the new species Sigambra nkossa (Annelida, Pilargidae), with an analysis of the distribution patterns of polychaetes associated with artificially hydrocarbon-enriched bottoms
Source: PeerJ. 2022 Oct 19;10:e13942. doi: 10.7717/peerj.13942 (PMC9587720; doi:10.7717/peerj.13942)
Supplement: Table S5 — D: difference; SD: Standard difference; CV: Critical value; P: probability; DDP: distance from drilling point; HYD: Total hydrocarbons; Ba: Barium; CS: coarse sand; S&C: silt and clay; OM: organic matter; PW: pore water; N: Nitrogen; P: Phosphorous; Snk: Sigambra nkossa sp. nov.; Sp: S. parva; Csp: Capitella sp. Pt: Paramphinome trionyx. Rsp: Raricirrus sp. Ob: Oxydromus berrisfordi. Ls: Lindaspio sebastiena. Amp: Ampharetidae sp. [file peerj-10-13942-s005.docx]

| DDP | D | SD | CV | P |  | Snk | D | SD | CV | P |
| --- | --- | --- | --- | --- | --- | --- | --- | --- | --- | --- |
| I vs III | 0.962 | 4.723 | 2.491 | **0.0002** |  | I vs III | 2.683 | 13.912 | 2.491 | **< 0.0001** |
| I vs II | 0.745 | 4.528 | 2.491 | **0.0004** |  | I vs II | 0.226 | 1.041 | 2.491 | 0.559 |
| II vs III | 0.217 | 0.943 | 2.491 | 0.619 |  | II vs III | 2.457 | 15.763 | 2.491 | **< 0.0001** |
| HYD |  |  |  |  |  | Sp |  |  |  |  |
| I vs III | 3.532 | 16.319 | 2.491 | **< 0.0001** |  | I vs III | 0.695 | 3.141 | 2.491 | **0.012** |
| I vs II | 1.267 | 5.189 | 2.491 | **< 0.0001** |  | I vs II | 0.304 | 1.921 | 2.491 | 0.154 |
| II vs III | 2.266 | 12.952 | 2.491 | **< 0.0001** |  | II vs III | 0.390 | 1.990 | 2.491 | 0.136 |
| Ba |  |  |  |  |  | Csp |  |  |  |  |
| I vs III | 1.662 | 9.421 | 2.491 | **< 0.0001** |  | I vs III | 2.162 | 8.429 | 2.491 | **< 0.0001** |
| I vs II | 0.487 | 2.445 | 2.491 | 0.055 |  | I vs II | 0.639 | 1.784 | 2.491 | 0.196 |
| II vs III | 1.176 | 8.245 | 2.491 | **< 0.0001** |  | II vs III | 1.524 | 4.800 | 2.491 | **0.0002** |
| CS |  |  |  |  |  | Pt |  |  |  |  |
| I vs III | 0.691 | 1.802 | 2.491 | 0.190 |  | I vs III | 1.077 | 4.413 | 2.491 | **0.0005** |
| I vs II | 0.124 | 0.364 | 2.491 | 0.930 |  | I vs II | 0.797 | 2.339 | 2.491 | **0.069** |
| II vs III | 0.567 | 2.064 | 2.491 | 0.118 |  | II vs III | 0.280 | 0.928 | 2.491 | 0.628 |
| S&C |  |  |  |  |  | Rsp |  |  |  |  |
| I vs III | 0.210 | 2.533 | 2.491 | **0.046** |  | I vs III | 0.886 | 3.473 | 2.491 | **0.0052** |
| I vs II | 0.110 | 1.849 | 2.491 | 0.175 |  | I vs II | 0.456 | 1.585 | 2.491 | 0.270 |
| II vs III | 0.100 | 1.363 | 2.491 | 0.375 |  | II vs III | 0.430 | 2.085 | 2.491 | 0.113 |
| PW |  |  |  |  |  | Ob |  |  |  |  |
| I vs III | 0.051 | 2.267 | 2.491 | 0.079 |  | I vs III | 0.787 | 4.835 | 2.491 | **0.0002** |
| I vs II | 0.002 | 0.088 | 2.491 | 0.996 |  | I vs II | 0.426 | 2.321 | 2.491 | 0.071 |
| II vs III | 0.049 | 1.947 | 2.491 | 0.147 |  | II vs III | 0.361 | 2.744 | 2.491 | **0.029** |
| OM |  |  |  |  |  | Ls |  |  |  |  |
| I vs III | 0.063 | 2.074 | 2.491 | 0.116 |  | I vs III | 0.642 | 4.042 | 2.491 | **0.001** |
| I vs II | 0.052 | 1.948 | 2.491 | 0.146 |  | I vs II | 0.531 | 2.964 | 2.491 | **0.017** |
| II vs III | 0.011 | 0.484 | 2.491 | 0.880 |  | II vs III | 0.111 | 0.866 | 2.491 | 0.666 |
| N |  |  |  |  |  | Amp |  |  |  |  |
| I vs III | 0.010 | 1.484 | 2.491 | 0.316 |  | I vs III | 1.171 | 4.893 | 2.491 | **0.0002** |
| I vs II | 0.004 | 0.409 | 2.491 | 0.912 |  | I vs II | 0.929 | 2.782 | 2.491 | **0.026** |
| II vs III | 0.006 | 0.738 | 2.491 | 0.743 |  | II vs III | 0.242 | 0.816 | 2.491 | 0.697 |
| P |  |  |  |  |  |  |  |  |  |  |
| I vs III | 0.191 | 2.477 | 2.491 | 0.051 |  |  |  |  |  |  |
| I vs II | 0.133 | 2.133 | 2.491 | 0.103 |  |  |  |  |  |  |
| II vs III | 0.058 | 0.668 | 2.491 | 0.784 |  |  |  |  |  |  |
