# Supplementary material for: Description of the new species Sigambra nkossa (Annelida, Pilargidae), with an analysis of the distribution patterns of polychaetes associated with artificially hydrocarbon-enriched bottoms
Source: PeerJ. 2022 Oct 19;10:e13942. doi: 10.7717/peerj.13942 (PMC9587720; doi:10.7717/peerj.13942)
Supplement: Table S6 — DC, dorsal cirri; LA, lateral antennae; MA, median antenna; TC, tentacular cirri; VC, ventral cirri; +/-, presence/absence of the character; ?, not reported. [file peerj-10-13942-s006.docx]

| **Species** | **No. of chaetigers** | **Length (mm)** | **Width (mm)** | **Body pigment** | **Eyes** | **MA>LA** | **Chaetiger reached by MA** | **Palps fused basally** | **Pharyngeal papillae** | **DC1/TC** | **DC2< following** | **DC>VC** | **VC2** | **Notopodial hooks from chaetiger** | **Notopodial spines from chaetiger** | **Notopodial capillaries*** | **Neuropodial capillaries*** | **Pointed neurochaetae dentate** | **Pointed neurochaetae pectinate** | **Bidentate neurochaetae** | **Type locality** |
| --- | --- | --- | --- | --- | --- | --- | --- | --- | --- | --- | --- | --- | --- | --- | --- | --- | --- | --- | --- | --- | --- |
| *S. bassi* (Hartman, 1947)** | up to 146 | 19–26 | 0.68–2 | - | - | >2 | 09-Dec | + | 14 | >1 | + | + | - | 10–15 | 12–15? | 0–1 | - | + | + | - | Lemon Bay, Florida, USA, Gulf of Mexico, intertidal |
| *S. bidentata* Britayev & Saphronova, 1981 | 45-60 | ? | ? | - | - | >1 | ? | - | 8 | 2 | + | + | - | 3–4 | - | 2 | - | + | - | + | Sea of Japan, 510–1730 m |
| *S. constricta* (Southern, 1921) | >120 | >23 | ? | - | - | 2 | 1–2 | - | 14 | 1.5 | + | + | - | 30–40 | - | 1 | - | + | - | - | Chilika Lake, India, Bay of Bengal, subtidal |
| *S. diazi* Salazar-Vallejo et al., 2019 | 70 | 9.5 | 1.5 | - | - | 2 | 2–3 | - | 14 | 2 | - | + | - | 4 | - | 13 | + | + | + | - | Lagoon of Chacopata, Venezuela, Southern Caribbean Sea, 1.5 m |
| *S. elegans* Britayev & Saphronova, 1981 | 101 | 16 | ? | - | - | 2 | ? | + | 13 | 1.5 | + | + | - | 1–16 | - | 1 | - | + | - | - | Sea of Japan, 6 m |
| *S. grubii* Müller, 1858*** | 82 | 16–26 | 2 | - | - | >1 | 2 | - | 14 | 1.5 | + | + | - | 9–21 | - | - | + | + | + | - | Santa Catarina Island, Brazil, Atlantic Ocean, intertidal |
| *S. hanaokai* (Kitamori, 1960) | 40–100 | 5–20 | 0.5–1 | - | - | 1.2–2.0 | 5–7 | - | 14 | >2.5 | + | + | - | 3–9 | - | 1 | + | + | + | - | Seto Inland Sea, Japan |
| *S. healyae* Gagaev, 2008 | 60+ | ? | 0.7 | reddish-brown spots, on median part of prostomium | + | 1.5 | ≈ 4 | - | 8 | 2 | ? | = or > | - | 4 | 20? | + | - | + | - | + | Canadian Basin, Arctic Ocean, 1825-1852 m |
| *S. hernandezi* Salazar-Vallejo et al., 2019 | 25–104 | 1.7–13 | 0.2–2 | - | - | 2 | 2–3 | - | 14 | 2 | + | + | - | 11–28 | - | - | + | + | + | - | Chetumal Bay, Mexico, NW Caribbean Sea, 1.5 m |
| *S. ligneroi* Salazar-Vallejo et al., 2019 | >49–90 | >17–33 | 4–5 | - | - | 2 | 1–2 | - | 8 | 2 | ? | > to ≈ | + | 27 | - | - | + | + | - | + | off Barcelona, Venezuela, Southern Caribbean Sea, 22 m |
| *S. magnuncus* Paterson & Glover, 2000 | 13–50 | 0.5–10.5 | 0.3 | - | - | 1.65 | beginning of 3 | + | 8 | 2 | + | + |  | 3 | - | 1–2 | + | - | - | - | Porcupine Abyssal Plain, Atlantic Ocean, 4844 m |
| *Sigambra nkossa* sp. nov. | 23–134 | 2.8–26 | 0.6–1.9 | faint blackish on dorsal and ventral prostomium and peristomium, and in antennae and tentacular cirri | - | 1.7 | 2 | - | 14 | 1.7 | + | + | - | 5(4–6) | 9(11) | 1 | + | + | + | - | off Republic of Congo, Gulf of Guinea, 180 m |
| *S. ocellata* (Hartmann-Schröder, 1959) | 25 | ca. 2 | ? | - | + (living material) | 2 | ? | + | 8 | 2 | - | - | - | 6–7 | - | 1 | + | - | - | - | Estero Jaltepeque, Gulf of Fonseca, El Salvador, Pacific Ocean, intertidal |
| *S. olivai* Salazar-Vallejo et al., 2019 | 128–152 | 15–24 | 2.8 | - | - | 2 | 2–3 | - | 15 | 2 | + | + | - | 30–39 | - | - | - | + | + | + | Nichupté Lagoon, Mexico, NW Caribbean Sea, 1.5 m. |
| *S. papagayu* Bamber in Muir & Bamber, 2008 | 122–126 | 17.25–22.81 | 1.5 | ant. region pale to white, following region reddish to brown | - | 1.75 | 5 | + | 8 | 1.5 | + | + | - | 3–5 | - | - | + | - | + | - | Conic Island Cave, Hong Kong, China, South China Sea, 15 m |
| *S. parva* (Day, 1963) | 30–75 | 6.75–12 | 0.35–0.8 | - | - | 1.5 | 5 | - | 14 | 1.5 | + | + | - | 4–5 | - | 1 | - | + | + | - | south coast of Cape Province, South Africa, Indian Ocean, 97 m |
| *S. pettiboneae* Hartmann-Schröder, 1979 | 66 | ? | ? | + | - | 2 | ? | - | 13 | >1 | + | + | - | 7–10 | - | 1 | - | + | - | - | Broome, N Western Australia, Australia, Indian Ocean, intertidal |
| *S. phuketensis* Licher & Westheide, 1997 | 46–85 | 4–9.3 | 0.5–0.75 | (+) spots in some specimens | - | 2 | 1–2 | - | 14 | 1.5 | + | + | - | 3–23 | - | 1 | - | + | - | - | Bang Tao Bay, Phuket Island, Thailand, Andaman Sea, 10 m |
| *S. qingdaoensis* Licher & Westheide, 1997 | 20–23 | 2.3 | 0.2 | - | - | 2 | 3 | + | 8 | 1.5 | + | + | - | 3–8 | - | 1 | - | + | - | - | Jiazhou Bay, near Qingdao, China, Yellow Sea |
| *S. robusta* (Ehlers, 1908) | 182 | 54 | ? | - | - | >1.5 | ≈ 6 | - | 15-16 | 2 | + | + | + | 43–70 | + | - | + | + | - | - | Tigres Bay, South Angola, Atlantic Ocean, about 20 m |
| *S. rugosa* Fauchald, 1972 | 80 | 14 | 2.5 | - | - | <1 | 1–2 | - | ? | <1.5 | + | + | - | 43–66 | - | - | + | + | ? | - | off Isla Espíritu Santo, Mexico, Gulf of California, 732 m |
| *S. setosa* Fauchald, 1972 | 60 | 14 | 3 | - | + | >1 | ? | + | 14 | >2 | - | + | - | 3–4 | - | 0–1 | + | + | - | - | off Isla Tortuga, Mexico, Gulf of California, 1823 m |
| *S. sundarbanensis* Bhowmik, Ghoshal, Salazar-Vallejo & Mandal, 2021 | 64 | 5.63 | 0.28 | - | - | 2,3 | 3–4 | ? | 14 | 0.5–0.9 | - | + | - | 8 | - | - | + | + | + | - | Sundarbans Estuarine System, India, 10–26 m |
| *S. tentaculata* (Treadwell, 1941) | 135/95 | 20-Nov | 0.5–0.62 | - | - | 1.5–2 | ≈ 6 | + | 14 | 1.5 | + | + | - | 4–5 | - | 0–1 | - | + | + | - | Long Island, NewYork, USA, Atlantic Ocean |
| *S. vargasi* Dean, 1999 | 50–136 | May-18 | 0.5–1 | ? | ? | 3 | 7 | ? | 8 | ? | ? | + | - | 15–17 | - | 1 | + | ? | ? | ? | Gulf of Nicoya, Costa Rica, Pacific Ocean, 18 m |
| *S. wassi* Pettibone, 1966 | 107–192 | 45–70 | 43589 | - | - | 1–1.3 | 1–2 | - | 14 | 2 | - | + | + | 23–31 | - | 1 | + | + | - | - | Chesapeake Bay, Virginia, USA, Atlantic Ocean, 11 m |

* According to our results, care should be taken when considering this character as, in *S. nkossa* sp. nov., these structures are protruding tips of notoaciculae.

** Based only in the specimens from Florida (Gulf of Mexico) by Hartman (1947), Pettibone (1966), and Wolf (1984).

*** Modified according to Salazar-Vallejo (1990) and Salazar-Vallejo et al. (2019).

**REFERENCES**

Hartman O. 1947. Polychaetous annelids. Part VIII. Pilargidae. *Allan Hancock Pacific Expeditions* 10:483-512

Pettibone MH. 1966. Revision of the Pilargidae (Annelida: Polychaeta), including descriptions of new species, and redescription of the pelagic Podarmus ploa Chamberlin (Polynoidae). *Proceedings of the United States National Museum* 118:155-208. DOI: 10.5479/si.00963801.118-3525.155

Salazar-Vallejo SI. 1990. Redescriptions of *Sigambra grubii* Müller, 1858 and Hermundura tricuspis Müller, 1858 from Brazil and designation of neotypes (Polychaeta: Pilargidae). *Journal of Natural History* 24:507-517. DOI: 10.1080/00222939000770351

Salazar-Vallejo SI, Rizzo AE, de León-González JÁ, Brauco KM. 2019. Four new Caribbean *Sigambra* species (Annelida, Pilargidae), and clarifications of three other *Sigambra* species. *ZooKeys* 893:21-50. DOI: 10.3897/zookeys.893.39594

Wolf PS. 1984. Family Pilargidae Saint-Joseph, 1899. In: Uebelacker JM, and Johnston PG, eds. *Taxonomic Guide to the Polychaetes of the Northern Gulf of Mexico, Volume 4*. Mobile, Alabama: Barry A.Vittor & Associates, Inc., 29.21-29.41.
