# Supplementary material for: Description of the new species Sigambra nkossa (Annelida, Pilargidae), with an analysis of the distribution patterns of polychaetes associated with artificially hydrocarbon-enriched bottoms
Source: PeerJ. 2022 Oct 19;10:e13942. doi: 10.7717/peerj.13942 (PMC9587720; doi:10.7717/peerj.13942)
Supplement: Table S7 — A. Size-dependent. B. Not varying with size. AVG, average; STD, standard deviation; Min, minimum; Max, maximum; Pearson, correlation coefficient; P, significance level; D, dorsal; V, ventral. [file peerj-10-13942-s007.docx]

| A | AVG | ± | STD | Min |  | Max | Pearson | P |
| --- | --- | --- | --- | --- | --- | --- | --- | --- |
| Number chaetigers | 87.000 | ± | 34.756 | 23 | – | 134 | – | – |
| Total length (µm) | 13941.429 | ± | 7243.994 | 2400 | – | 26000 | 0.979 | <0.0001 |
| Width chaetiger 15 with parapodia (µm) | 1384.524 | ± | 411.321 | 570 | – | 1900 | 0.930 | <0.0001 |
| Width chaetiger 15 without parapodia (µm) | 575.714 | ± | 186.167 | 190 | – | 850 | 0.896 | <0.0001 |
| Dry weight (mg) | 1.396 | ± | 0.428 | 1 | – | 2 | 0.981 | <0.0001 |
| Length of prostomium (µm) | 365.714 | ± | 102.887 | 175 | – | 550 | 0.789 | <0.0001 |
| Width of prostomium (µm) | 553.810 | ± | 147.088 | 240 | – | 770 | 0.897 | <0.0001 |
| Length median antenna (µm) | 834.524 | ± | 275.218 | 260 | – | 1280 | 0.932 | <0.0001 |
| Length lateral antennae (µm) | 486.905 | ± | 169.422 | 175 | – | 825 | 0.861 | <0.0001 |
| D peristomial cirri (µm) | 745.714 | ± | 287.269 | 185 | – | 1280 | 0.888 | <0.0001 |
| V peristomial cirri (µm) | 536.429 | ± | 202.214 | 170 | – | 780 | 0.894 | <0.0001 |
| Length 1st D cirri (µm) | 1237.143 | ± | 454.328 | 320 | – | 1985 | 0.918 | <0.0001 |
| Length 2nd D cirri (µm) | 269.286 | ± | 111.100 | 85 | – | 450 | 0.880 | <0.0001 |
| Length 3rd D cirri (µm) | 376.905 | ± | 160.113 | 120 | – | 650 | 0.816 | <0.0001 |
| Length 1st V cirri (µm) | 229.048 | ± | 97.206 | 70 | – | 380 | 0.911 | <0.0001 |
| Length anal cirri (µm) | 1240.476 | ± | 492.887 | 440 | – | 2460 | 0.801 | <0.0001 |
| Starting chaetiger for protruding acicular tips | 33.905 | ± | 31.868 | 0 | – | 108 | 0.780 | <0.0001 |
| B | | | | | | | | |
| Starting chaetiger for notopodial hooks | 5.143 | ± | 0.359 | 5 | – | 6 | -0.038 | 0.868 |
| Starting chaetiger for protruding acicular tips | 9.380 | ± | 0.805 | 9 | – | 12 | 0.177 | 0.476 |
| Length ratio for antennae (middle / lateral) | 1.738 | ± | 0.288 | 1.18 | – | 2.34 | 0.122 | 0.599 |
| Length ratio for peristomial cirri (D / V) | 1.394 | ± | 0.189 | 1.09 | – | 1.75 | 0.086 | 0.709 |
| Length ratio for cirri (D peristomial / 1st D) | 1.679 | ± | 0.164 | 1.41 | – | 1.97 | -0.063 | 0.787 |
| Second V cirri (µm) | – |  | – | – |  | – | – | – |
| Length ratio for cirri (1st D / 1st V) | 4.709 | ± | 0.730 | 3.75 | - | 6.24 | -0.120 | 0.605 |
